# Diagnostic Performance of Galectin-1 and Mucin-1 in Uterine Tumors: A Case–Control Study

**DOI:** 10.3390/cimb48090948

**Published:** 2026-09-16

**Authors:** Paraskevi Karioti, Aikaterini Sidera, Ioannis Tsakiridis, Areti Kourti, Georgios Karavas, Themistoklis Dagklis, Apostolos Mamopoulos, Apostolos Athanasiadis, Kali Makedou, Ioannis Kalogiannidis

**Affiliations:** 1 Third Department of Obstetrics and Gynecology, School of Medicine, Faculty of Health Sciences, Aristotle University of Thessaloniki, Konstantinoupoleos 49, 54642 Thessaloniki, Greece; pkarioti@auth.gr (P.K.); k.sidera@erasmusmc.nl (A.S.); gkaravas@auth.gr (G.K.); dagklis@auth.gr (T.D.); amamop@auth.gr (A.M.); apathana@auth.gr (A.A.); ikalogia@auth.gr (I.K.); 2 Laboratory of Biological Chemistry, School of Medicine, Aristotle University of Thessaloniki, 54124 Thessaloniki, Greece; aretikourti@auth.gr; 3 Laboratory of Biochemistry, AHEPA University Hospital, School of Medicine, Aristotle University of Thessaloniki, 54636 Thessaloniki, Greece; kmakedou@auth.gr

**Keywords:** uterine sarcoma, leiomyoma, STUMP, galectin-1, mucin-1, biomarkers, preoperative diagnosis

## Abstract

Background and Objectives: Uterine leiomyomas are common benign tumors, whereas uterine sarcomas are rare but aggressive malignancies. Preoperative differentiation among leiomyoma, smooth muscle tumor of uncertain malignant potential (STUMP), and uterine sarcoma remains a major clinical challenge, with diagnosis often established only after histopathological evaluation. The identification of reliable circulating biomarkers could improve preoperative risk stratification and guide surgical decision-making. This study aimed to evaluate the usefulness of galectin-1 and mucin-1 in the diagnosis of uterine tumors. Materials and methods: This prospective, single-center case–control study was conducted at the Third Department of Obstetrics & Gynecology, School of Medicine, Faculty of Health Sciences, Aristotle University of Thessaloniki, Greece, between 2013 and 2024. Serum galectin-1 and mucin-1 levels were measured preoperatively using ELISA and electrochemiluminescence immunoassay (ECLIA), respectively. Group comparisons were performed using non-parametric tests. Diagnostic performance was evaluated using receiver operating characteristic (ROC) curve analysis. Multivariable logistic regression was used to assess independent associations with sarcomas/STUMP status. Results: The study population comprised 156 women (49 healthy controls, 90 patients with uterine myomas, 8 with STUMP and 9 patients with uterine sarcomas) treated during the study period. Galectin-1 differed significantly across the four histological groups (*p* < 0.001), with higher concentrations in uterine sarcoma than in healthy controls (*p* < 0.001) and leiomyoma (*p* = 0.002). MUC1 also differed overall (*p* = 0.031), with higher concentrations in uterine sarcoma than in leiomyoma (adjusted *p* = 0.031). In the primary leiomyoma versus sarcoma/STUMP ROC analysis, galectin-1 showed modest discrimination (AUC 0.693, 95% CI 0.559–0.826), while MUC1 showed weaker discrimination (AUC 0.618, 95% CI 0.468–0.768). Conclusions: Serum galectin-1 may have potential as an adjunctive biomarker in the preoperative assessment of uterine tumors, but its modest discrimination and unstable cutoff-dependent performance may preclude its use as a standalone diagnostic test. MUC1 showed limited diagnostic utility in the primary clinical comparison. Given the clinical implications of misdiagnosis, particularly regarding surgical management, further validation in larger cohorts and multimarker approaches are warranted.

## 1. Introduction

Uterine tumors are a frequent finding in gynecological practice, with uterine myomas representing the most common tumors, affecting up to 70% of women of reproductive age [1,2]. In contrast, uterine sarcomas are rare but highly aggressive malignancies, accounting for about 3% of uterine cancers [3,4]. In addition, uterine smooth muscle tumors of uncertain malignant potential (STUMPs) represent a diagnostically challenging intermediate category, characterized by histopathological features that overlap between benign leiomyomas and malignant leiomyosarcomas failing to meet the strict criteria for either, making them a “gray zone” entity [5]. Women with uterine tumors are usually asymptomatic but may experience symptoms like abnormal uterine bleeding (AUB), dysmenorrhea, a feeling of pressure in the pelvis, bladder or bowel symptoms and even challenges with fertility [6,7,8,9,10].

Despite their distinct biological behavior, differentiating benign myomas from uterine sarcomas preoperatively remains a significant clinical challenge [11]. Current diagnostic approaches rely on clinical evaluation and imaging modalities, including ultrasound and magnetic resonance imaging (MRI) [12]. However, substantial overlap in radiological and clinical features frequently limits their diagnostic accuracy [13]. Consequently, definitive diagnosis is most often established postoperatively through histopathological examination of the surgical specimen [14]. Management strategies for uterine masses typically include conservative or surgical approaches, with myomectomy or hysterectomy being the main treatment options depending on patient age, symptom burden, and reproductive ability [15,16]. In cases where malignancy is not suspected, minimally invasive techniques such as morcellation during a laparoscopic approach may be employed, which may inadvertently worsen prognosis by facilitating tumor dissemination [17,18,19]. Given the absence of reliable methods to exclude malignancy preoperatively, and although the risk remains low, any biomarker that improves the probability of ruling in malignancy could affect surgical approach and tissue extraction strategies (en bloc removal vs. morcellation). This is consistent with both FDA recommendations advocating for contained morcellation and careful patient selection [20], as well as current existing guidance on uterine sarcomas, which emphasizes appropriate oncologic surgical planning when malignancy is suspected [16,21,22].

Galectin-1, a β-galactoside-binding lectin, has been implicated in key processes of tumorigenesis, including cell proliferation, angiogenesis, immune evasion, and tumor–stromal interactions [23]. Increased expression of galectin-1 has been reported in various malignancies and has been associated with tumor progression and poor clinical outcomes [24]. Similarly, mucin-1 (MUC1), a transmembrane glycoprotein expressed on epithelial surfaces, is frequently overexpressed and aberrantly glycosylated in malignant tissues, contributing to tumor invasion, metastasis, and resistance to apoptosis [25]. Elevated circulating levels of MUC1 have been described in several cancers, suggesting its potential role as a diagnostic and prognostic biomarker [26]. Although these biomarkers have been extensively studied in other cancer types, their role in the context of uterine tumors remains insufficiently explored. Importantly, neither marker is tumor-specific. Galectin-1 also participates in tissue repair and fibrotic processes, while circulating MUC1-derived markers such as CA15-3 may be elevated in non-malignant conditions, including inflammatory, pulmonary-fibrotic, and hepatic disorders. Such non-oncological influences may reduce specificity and should be considered when interpreting these proteins as candidate biomarkers in the differential diagnosis of uterine masses [23,27].

Given the morbidity of delayed diagnosis and the dissemination risk associated with morcellation in occult malignancy, this study aimed to investigate the serum levels of galectin-1 and MUC1 in women with uterine tumors.

## 2. Material & Methods

### 2.1. Study Design and Population

This prospective, single-center case–control study was conducted at the Third Department of Obstetrics & Gynecology, School of Medicine, Faculty of Health Sciences, Aristotle University of Thessaloniki, Greece, between 2013 and 2024. The primary objective was to evaluate the potential of serum galectin-1 and MUC1 to discriminate between uterine leiomyoma and sarcoma/STUMP. Secondary objectives included comparing circulating biomarker concentrations across healthy controls, leiomyoma, STUMP, and uterine sarcoma groups and exploring associations with selected clinical and demographic characteristics.

The study group comprised women with uterine myomas, uterine sarcomas, and STUMPs who underwent abdominal surgery, with definitive diagnosis established by histopathological examination. Given the extended study period (2013–2024), histopathological diagnoses were established according to the diagnostic standards applicable at the time of assessment; a uniform retrospective re-review of all specimens according to the most recent WHO classification was not performed. For the main diagnostic analyses, uterine sarcomas and STUMPs were combined because of the limited number of cases in each subgroup. Clinical and demographic characteristics included age, body mass index (BMI), menopausal status, smoking and alcohol consumption, as well as medical history including hypertension, thyroid disease, diabetes mellitus, and endometriosis, as well as obstetric history. Tumor-related characteristics and management were additionally documented, including tumor morphology, surgical procedure, histological findings, and additional treatments. The control group consisted of asymptomatic women with no evidence of uterine pathology, as confirmed by transvaginal pelvic ultrasound before enrollment. Specific exclusion criteria regarding inflammatory, autoimmune, or other systemic diseases were not predefined in the study protocol. Galectin-1 and MUC1 were measured preoperatively in all participants. Histopathology reports set the final diagnosis on the nature of the tumor removed, after the surgical removal.

### 2.2. Biomarker Measurements

Serum galectin-1 and MUC1 were measured in all participants. In women undergoing surgery, blood samples were collected preoperatively; in healthy controls, blood samples were collected at study enrollment. Samples were processed according to institutional protocols; serum was separated by centrifugation and stored at −80 °C until analysis. Galectin-1 serum concentrations were measured using a commercially available Boster Picokine Human LGALS1 Pre-Coated ELISA kit by Boster Biological Technology (Pleasanton, CA, USA). MUC1 was assayed in serum on the automated analyzer e801 of the analytical system COBAS 8000 (Roche Diagnostics, Mannheim, Germany) with electrochemiluminescence sandwich immunoassay (ECLIA), using monoclonal antibodies. Consistency of all preanalytical procedures across the full 2013–2024 study period was not systematically documented. Assay-specific analytical performance characteristics, including coefficients of variation and limits of detection/quantification, were not available in the archived study records and therefore could not be retrospectively reported. Information regarding preoperative medical treatment, including GnRH agonists or other hormonal therapies, was not systematically recorded. All biomarker data were recorded in a unified dataset and checked for completeness and outliers.

### 2.3. Statistical Analysis

Descriptive statistics were used to summarize demographic, clinical, tumor-related, and biomarker characteristics. Continuous variables were assessed for normality using the Shapiro–Wilk test and, given their predominantly non-normal distributions, they are presented as median and interquartile range (IQR), while categorical variables were expressed as counts and percentages. Differences between the three groups (healthy controls, myomas, and sarcomas/STUMPs) were evaluated using the Kruskal–Wallis test, followed by pairwise comparisons using the Wilcoxon rank-sum test with Bonferroni correction. Categorical variables were compared using Pearson’s chi-squared test or Fisher’s exact test, depending on expected cell counts. Biomarker concentrations were compared across four histological groups (healthy controls, leiomyoma, STUMP, and uterine sarcoma) using the Kruskal–Wallis test followed by Dunn’s post hoc test with Holm adjustment for multiple comparisons. Biomarker distributions were visualized using jitter plots displaying individual observations.

Receiver operating characteristic (ROC) curve analysis was performed to evaluate the diagnostic performance of galectin-1 and MUC1 in distinguishing between study groups. The clinically relevant comparison between leiomyoma and the combined sarcoma/STUMP group was considered the primary ROC analysis, whereas comparisons involving healthy control and myoma groups were considered secondary exploratory analyses. STUMP and uterine sarcoma cases were combined because of the limited number of cases in each subgroup and the resulting insufficient statistical power for separate ROC analyses; this grouping does not imply biological or histopathological equivalence. The area under the ROC curve (AUC) with corresponding 95% confidence intervals (CIs), optimal cutoff values determined using Youden’s index, and corresponding sensitivity and specificity were calculated. For the primary comparison, positive and negative predictive values (PPV and NPV) were also calculated at the Youden-derived cutoff. Exact 95% binomial confidence intervals were calculated for sensitivity, specificity, PPV, and NPV. Because PPV and NPV depend on disease prevalence and the case–control sampling fraction does not represent population prevalence, these values were interpreted only within the study sample. No formal adjustment for multiplicity was applied to the secondary ROC analyses; consequently, these findings were considered exploratory and interpreted cautiously. Internal validation of the primary ROC analysis (leiomyoma vs. sarcoma/STUMP) was performed using 2000 stratified bootstrap iterations. In each iteration, cases and controls were resampled separately with replacement, the optimal cutoff was determined in the bootstrap sample using Youden’s index, and its performance was evaluated in the corresponding out-of-bag observations. Mean out-of-bag AUC, sensitivity, specificity, and cutoff values were calculated together with percentile-based 95% bootstrap intervals. Separate exploratory multivariable logistic regression models were used to evaluate biomarker associations with sarcoma/STUMP status after adjustment for age and menopausal status.

Due to the small number of cases within individual uterine sarcoma histological subtypes, subtype-specific inferential analyses were not performed. Statistical analysis was performed using R (version 4.4.1). A two-sided *p*-value < 0.05 was considered statistically significant.

### 2.4. Ethics

The study was conducted in accordance with the Declaration of Helsinki and was approved by the Institutional Review Board/Ethics Committee of Aristotle University of Thessaloniki (approval number: 1.75/21.11.2018). Written informed consent was obtained from all participants before inclusion in the study and no incentives were provided.

## 3. Results

### 3.1. Study Population

A total of 156 women were included in the analysis, comprising 49 healthy controls, 90 patients with uterine leiomyomas, 8 patients with STUMP, and 9 patients with uterine sarcomas. Baseline demographic and clinical characteristics are summarized in Table 1. Age and BMI were comparable across the three main analytical groups (*p* = 0.529 and *p* = 0.811, respectively). Significant between-group differences were observed in menopausal status (*p* < 0.001), alcohol consumption (*p* = 0.006), and hypertension (*p* = 0.006). No significant differences were observed with respect to parity, smoking, thyroid disease, diabetes mellitus, or endometriosis.

### 3.2. Histopathological Characteristics and Surgical Management

Histopathological characteristics are summarized in Table 2. The myoma group consisted predominantly of leiomyomas, with a small proportion of cases showing coexisting adenomyosis or other benign findings. The sarcoma/STUMP group comprised 8 STUMPs and 9 uterine sarcomas, including leiomyosarcoma, carcinosarcoma, endometrial stromal sarcoma, adenosarcoma, and one other histological diagnosis.

Surgical management varied between groups. Myomectomy was most frequently performed in patients with myomas, whereas hysterectomy-based procedures were more common in the sarcoma/STUMP group (Table 3).

### 3.3. Biomarker Levels

**Figure 1:**
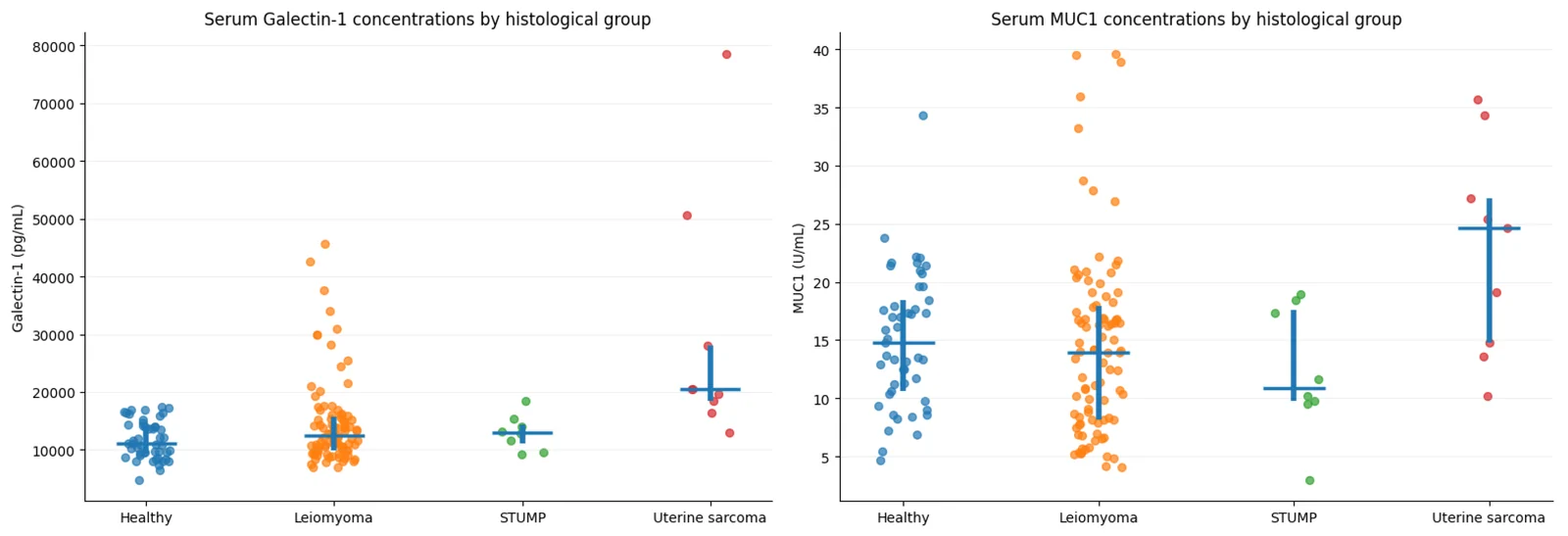
Distribution of galectin-1 and mucin-1 levels across study groups (boxplot with jitter).

Serum biomarker concentrations differed across the study groups. Galectin-1 showed a significant overall difference among healthy controls, leiomyoma, STUMP, and uterine sarcoma groups (Kruskal–Wallis *p* < 0.001; Table 4). Dunn’s post hoc pairwise comparisons with Holm adjustment demonstrated significantly higher galectin-1 concentrations in uterine sarcoma than in healthy controls (adjusted *p* < 0.001) and leiomyoma (adjusted *p* = 0.002). No significant difference was observed between leiomyoma and STUMP (adjusted *p* = 0.949), while the difference between STUMP and uterine sarcoma did not reach statistical significance. MUC1 concentrations also differed significantly across the four groups (Kruskal–Wallis *p* = 0.031). In post hoc analysis, MUC1 concentrations were significantly higher in uterine sarcoma than in leiomyoma (adjusted *p* = 0.031), whereas the remaining pairwise comparisons were not statistically significant. Jitter plots showed the distributions of individual biomarker values across the four histological groups (Figure 1).

### 3.4. Diagnostic Performance of Biomarkers

**Figure 2:**
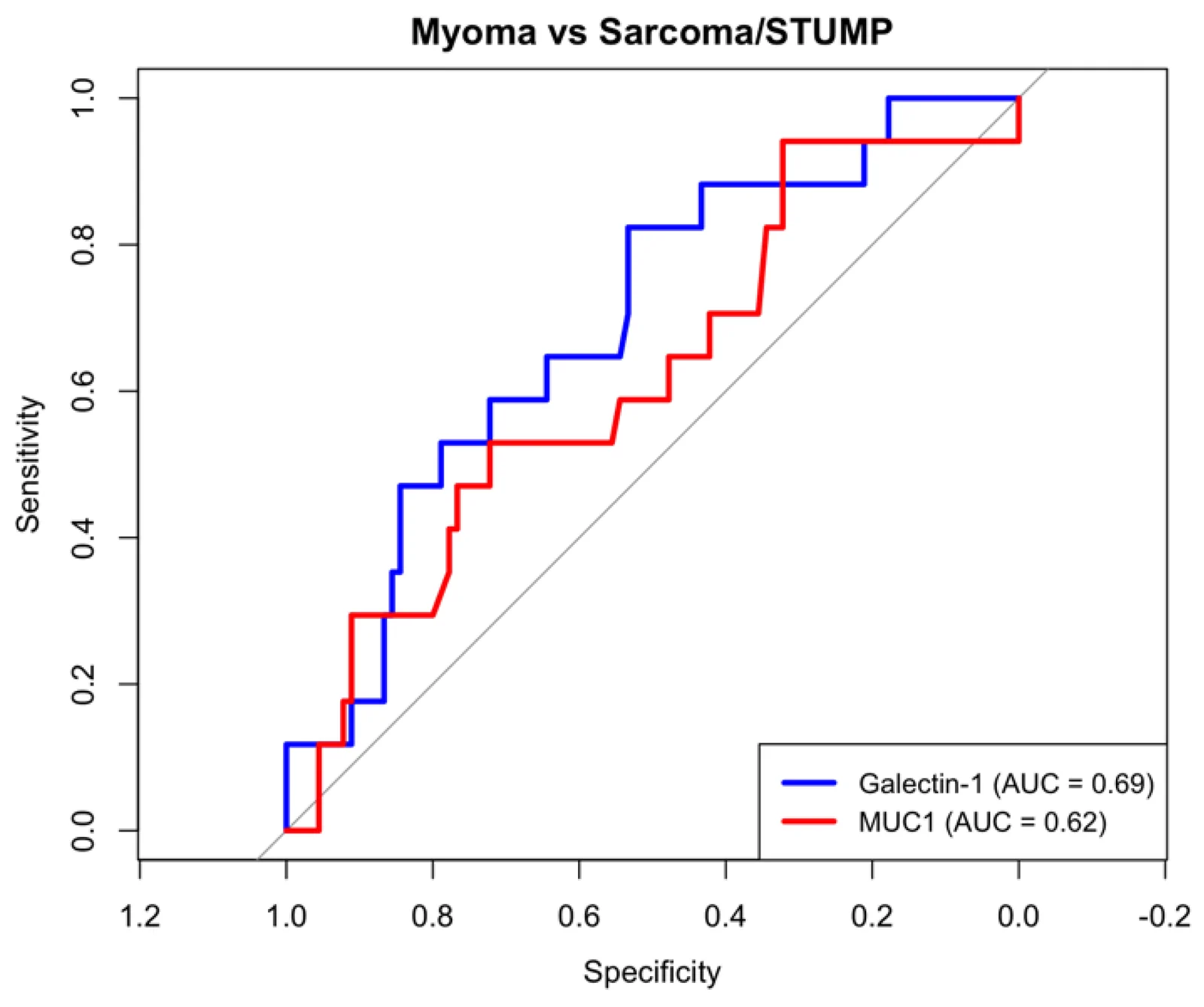
Receiver operating characteristic (ROC) curve of galectin-1 and MUC1 for differentiating uterine sarcoma/STUMP from myoma.

**Figure 3:**
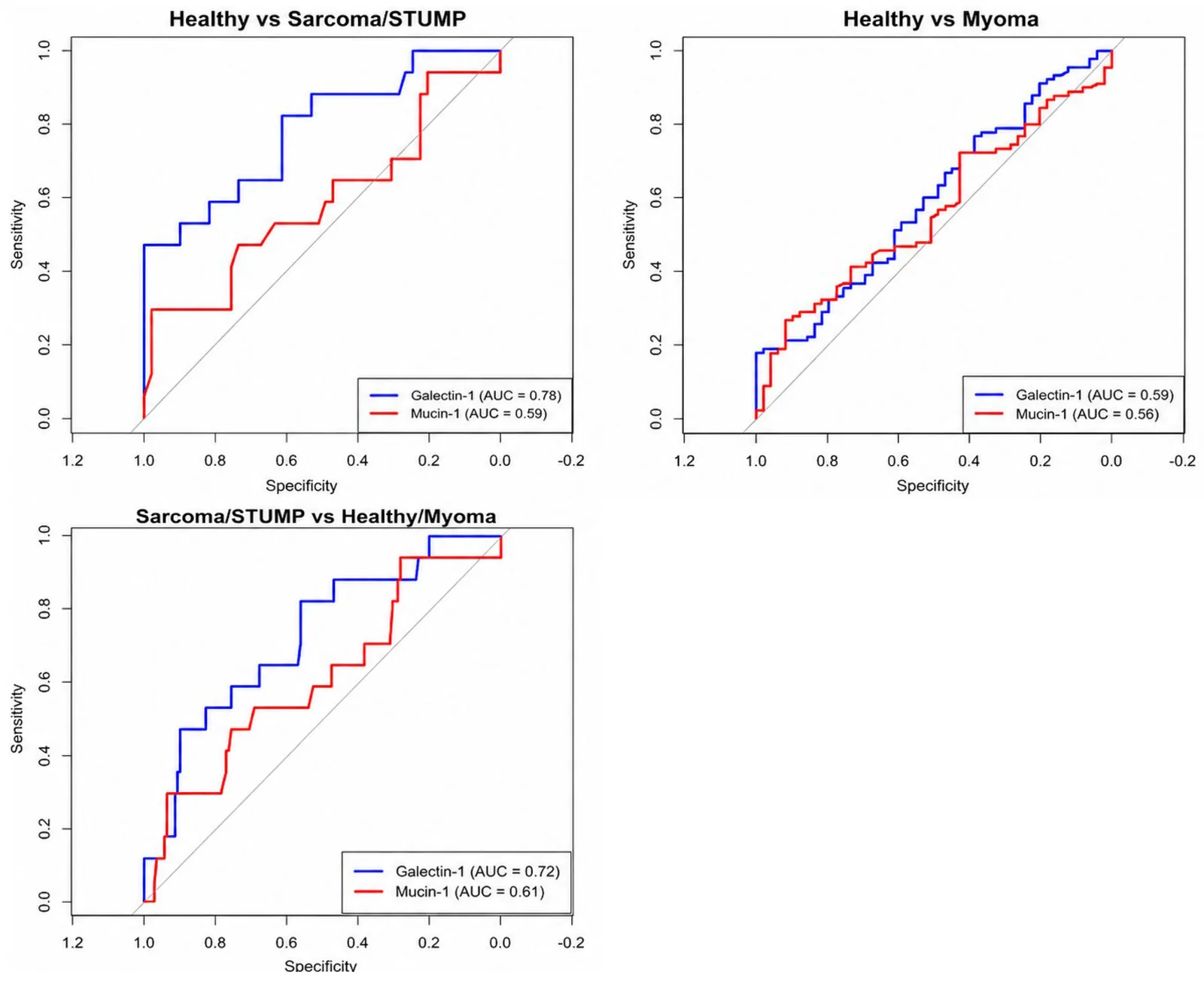
Receiver operating characteristic (ROC) curve of galectin-1 and MUC1 in distinguishing healthy controls from myoma and sarcoma/STUMP groups. The grey line is the midline.

Receiver operating characteristic (ROC) curve analysis was performed to evaluate the diagnostic performance of galectin-1 and MUC1 across the study groups (Table 5). In the primary comparison between myoma and sarcoma/STUMP, galectin-1 showed modest discriminative ability (AUC 0.693, 95% CI 0.559–0.826). At the optimal cutoff of 12,866.4 pg/mL, sensitivity was 82.4% (95% CI 56.6–96.2), specificity 53.3% (42.5–63.9), PPV 25.0% (14.4–38.4), and NPV 94.1% (83.8–98.8). MUC1 showed weaker performance (AUC 0.618, 95% CI 0.468–0.768); at the optimal cutoff of 9.31 U/mL, sensitivity was 94.1% (71.3–99.9), specificity 32.2% (22.8–42.9), PPV 20.8% (12.4–31.5), and NPV 96.7% (82.8–99.9) (Figure 2). Galectin-1 performed better in distinguishing sarcoma/STUMP from healthy controls (AUC 0.785, 95% CI 0.649–0.920); at the optimal cutoff of 17,928 pg/mL, specificity was 100.0% and sensitivity 47.1%. MUC1 showed poor discrimination in this comparison (AUC 0.588, 95% CI 0.412–0.763). In secondary analyses, galectin-1 showed limited discrimination between healthy controls and myoma patients (AUC 0.594, 95% CI 0.496–0.692) and moderate discrimination between sarcoma/STUMP and the combined healthy/myoma group (AUC 0.725, 95% CI 0.597–0.853). MUC1 demonstrated weak discriminatory performance in both secondary comparisons (Figure 3).

Internal validation of the primary ROC analysis was performed using 2000 stratified bootstrap iterations with out-of-bag evaluation. For galectin-1, the mean internally validated AUC was 0.697 (95% bootstrap interval 0.506–0.875), while the mean sensitivity and specificity were 60.7% and 61.0%, respectively. For MUC1, the corresponding validated AUC was 0.620 (95% bootstrap interval 0.421–0.826), with mean sensitivity and specificity of 57.3% and 56.2%, respectively (Appendix A Table A1).

### 3.5. Exploratory Multivariable Analysis

Separate exploratory multivariable logistic regression models were used to evaluate biomarker associations with sarcoma/STUMP status after adjustment for age and menopausal status (Table 6). Higher galectin-1 concentrations were associated with sarcoma/STUMP status (OR 1.07 per 1000 pg/mL increase; 95% CI 1.02–1.13; *p* = 0.007). In the galectin-1 model, age and menopausal status were not significantly associated with sarcoma/STUMP status. MUC1 was not significantly associated with sarcoma/STUMP status in the corresponding adjusted model (OR 1.05 per 1 U/mL increase; 95% CI 0.98–1.12; *p* = 0.145).

## 4. Discussion

The present study evaluated serum galectin-1 and MUC1 in women with leiomyoma, STUMP, uterine sarcoma, and healthy controls. Galectin-1 concentrations differed significantly across the four histological groups, with the highest median levels observed in uterine sarcoma and significant pairwise differences between uterine sarcoma and both healthy controls and leiomyoma. MUC1 also differed overall across the four groups, with higher concentrations in uterine sarcoma than in leiomyoma, although its discriminatory performance in the primary leiomyoma versus sarcoma/STUMP ROC analysis was weak. In exploratory adjusted analysis, higher galectin-1 concentrations were associated with sarcoma/STUMP status.

Differentiating between benign uterine fibroids from malignant uterine sarcomas and intermediate entities such as STUMP remains one of the most challenging aspects of gynecologic practice [28]. Although myomas are highly prevalent and benign, uterine sarcomas are rare but aggressive malignancies with poor prognosis [11]. STUMP is a rare and heterogeneous diagnosis positioned between leiomyoma and leiomyosarcoma in classification systems, typically established only after postoperative histopathologic evaluation using criteria centered on cytologic atypia, mitotic activity, and coagulative tumor necrosis [29]. Despite their typically low malignant potential, these tumors may occasionally recur or metastasize [30]. Gupta et al. recently proposed updated diagnostic criteria for STUMP, based on WHO guidelines and multicenter experience; these include borderline or difficult-to-define tumor necrosis, atypia with mitotic activity near malignant thresholds, high mitotic counts, focal coagulative necrosis in otherwise benign-appearing tumors, intermediate cytologic features, myometrial invasion without overt malignancy, and atypical mitotic figures in the absence of other malignant characteristics [31].

Preoperative differentiation is often limited by overlapping clinical presentation and imaging characteristics, and definitive diagnosis is frequently established only after surgical removal and histopathological examination [32]. From a clinical standpoint, the need for improved preoperative risk stratification is underscored by the well-recognized, low but consequential risk of occult uterine sarcoma at surgery for presumed fibroids and the potential for iatrogenic dissemination with power morcellation [33]. The FDA’s 2014 Safety Communication discouraged the use of laparoscopic power morcellation in most women undergoing hysterectomy or myomectomy for fibroids, and updated 2020 guidance recommends that, when morcellation is appropriate, it should be performed only with a containment system and in carefully selected patients [20,34]. Therefore, the identification of reliable, non-invasive biomarkers capable of improving preoperative risk stratification remains an important unmet clinical need.

Multiple studies across malignancies suggest that circulating galectin-1 can reflect tumor aggressiveness and microenvironmental remodeling, but diagnostic performance is context-dependent; as a β-galactoside-binding lectin, galectin-1 is involved in several processes central to tumor development and progression, including modulation of the tumor microenvironment, angiogenesis, and immune evasion [35,36]. In ovarian tumor cohorts, serum galectin-1 measured by ELISA has shown excellent discrimination in some settings (e.g., AUC 0.936 with a reported cutoff of 15.9 ng/mL achieving 88.9% sensitivity and 93% specificity), while other programs—particularly those focused on high-grade serous ovarian carcinoma—reported minimal case–control separation in plasma despite stromal galectin-1 being prognostically informative in tissue [37,38]. This variability is consistent with galectin-1 acting as a stromal/immune biomarker: in pancreatic cancer, where galectin-1 is strongly linked to desmoplastic stroma, plasma galectin-1 is elevated versus healthy controls but can also rise in benign inflammatory fibrosis (chronic pancreatitis), and performance improves when combined with an established marker (CA19-9) [39,40,41]. Published tissue-based analyses have reported lower galectin-1 expression in leiomyosarcomas compared to leiomyomas [42]. This finding is not directly comparable with the present study because we measured circulating serum galectin-1 rather than tumor-tissue expression. In our cohort, galectin-1 was higher in uterine sarcomas than in myomas and controls and remained associated with sarcoma after adjustment for age and menopausal status.

From a clinical perspective, the most relevant diagnostic challenge is the preoperative distinction between presumed leiomyoma and sarcoma/STUMP. In this setting, galectin-1 demonstrated only modest discrimination, with an AUC of 0.69. Although performance was higher when sarcoma/STUMP cases were compared with healthy controls, this contrast is less representative of the real-world diagnostic setting. Internal bootstrap validation yielded an AUC estimate similar to the apparent analysis, but cutoff-dependent sensitivity and specificity showed considerable variability. Taken together, these findings do not support the use of galectin-1 as a standalone diagnostic test. Its potential value may instead lie as an adjunct to existing clinical and imaging assessment, provided that its performance is confirmed in larger, clinically representative cohorts.

Circulating MUC1 levels are highly epitope- and assay-dependent and can differ between benign and malignant disease but often show high inter-individual variability and limited differential diagnostic utility, whereas tumor-epitope-specific MUC1 assays can achieve higher discrimination [43]. Furthermore, variations in glycosylation and the release of soluble MUC1 fragments into circulation may influence its detectability and clinical utility as a serum biomarker [26]. Consistent with these findings, our study demonstrated significant overall differences in circulating MUC1 levels between groups, with higher concentrations in uterine sarcoma than in myoma; however, MUC1 showed weak discrimination in the primary myoma versus sarcoma/STUMP comparison. Although MUC1 plays a well-established role in tumor progression in epithelial malignancies, its expression in mesenchymal tumors such as uterine sarcomas may follow different biological patterns [44]. In ovarian tumor patients, CA 15-3 and CA 27.29 (MUC-derived assays) can be higher in malignancy but show substantial overlap and high variability [45]. Similarly, MUC1 can behave paradoxically (lower median in ovarian cancer than benign disease) and achieves only modest sensitivity at high specificity (AUC 0.784 with 37% sensitivity at 95% specificity) [46]. In uterine tumors, the role of MUC1 appears to be less straightforward; while MUC1 is associated with the presence of tumor tissue, it may not reliably distinguish between benign and malignant uterine lesions at the tissue level [42].

Strengths of this study include preoperative serum sampling and histopathological confirmation of tumor diagnoses. Several limitations should be acknowledged. First, the small numbers of STUMP (n = 8) and uterine sarcoma (n = 9) cases limited statistical power, and the number of covariates relative to outcome events introduced a risk of overfitting; accordingly, adjusted analyses should be regarded as exploratory. Second, the sarcoma/STUMP group was histologically heterogeneous, limiting subtype-specific conclusions. The prolonged accrual period (2013–2024) may also have introduced temporal heterogeneity, because diagnostic and clinical practices evolved over time, and cases were not uniformly re-evaluated according to the most recent WHO classification. Another limitation concerns the control group: although controls were asymptomatic and had normal transvaginal ultrasound findings, predefined exclusion criteria for inflammatory, autoimmune, or other systemic conditions were unavailable, and controls were not spectrum-matched to women presenting with uterine masses, introducing potential spectrum bias. Comparisons with healthy controls should therefore be interpreted primarily as reference information rather than estimates of real-world diagnostic performance. Preanalytical factors and preoperative hormonal treatments were not systematically documented. Menopausal status, alcohol consumption, and hypertension differed among study groups. Although age and menopausal status were included in exploratory adjusted models, the limited number of sarcoma/STUMP events precluded stable adjustment for a larger set of covariates. Residual confounding related to alcohol use, hypertension, unrecorded inflammatory conditions, other comorbidities, and unavailable tumor-size information cannot be excluded. Internal bootstrap validation yielded AUC estimates broadly consistent with the apparent analyses; however, cutoff-dependent sensitivity and specificity showed considerable variability, and the derived thresholds should not be regarded as clinically validated cutoffs. Finally, the single-center design, absence of external validation, and potential residual confounding limit generalizability.

Future studies could integrate serum proteomic profiling with paired tumor-tissue expression analyses to determine whether circulating biomarker signals reflect tumor production, stromal responses, or systemic host processes, and to identify multimarker panels with greater discriminatory performance.

## 5. Conclusions

Our findings suggest that serum galectin-1 may have potential as an adjunctive biomarker in the preoperative assessment of uterine tumors, whereas MUC1 showed limited diagnostic utility in the primary clinical comparison. The modest discriminatory performance of galectin-1 between myoma and sarcoma/STUMP and the instability of cutoff-dependent estimates preclude its use as a standalone diagnostic test. Larger prospective studies involving clinically representative populations and external validation are required to determine its potential role within multimodal diagnostic strategies.

## Figures and Tables

**Table 1:** Baseline demographic and clinical characteristics of the study population.

Variable	Healthy (n = 49)	Myoma (n = 90)	Sarcoma/STUMP (n = 17)	*p*-Value
**Age (years)**	48 (13)	45 (9.75)	48 (11)	0.529
**BMI (kg/m^2^)**	26 (7)	27 (5.95)	26 (4)	0.811
**Menopausal status**				**<0.001** *
Postmenopausal	20 (40.8%)	11 (12.2%)	5 (29.4%)	
Premenopausal	29 (59.2%)	79 (87.8%)	12 (70.6%)	
**Parity**				0.369
Nulliparous	14 (28.6%)	22 (24.4%)	7 (41.2%)	
Parous (≥1)	35 (71.4%)	68 (75.6%)	10 (58.8%)	
**Smoking**	13 (26.5%)	34 (37.8%)	5 (29.4%)	0.379
**Alcohol consumption**	24 (49.0%)	21 (23.3%)	4 (23.5%)	**0.006** *
**Hypertension**	11 (22.4%)	6 (6.7%)	5 (29.4%)	**0.006** *
**Thyroid disease**	8 (16.3%)	17 (18.9%)	3 (17.6%)	0.931
**Diabetes mellitus**	1 (2.0%)	3 (3.3%)	1 (5.9%)	0.737
**Endometriosis**	0 (0.0%)	3 (3.4%)	0 (0.0%)	0.317

Data are presented as median (interquartile range [IQR]) for continuous variables and as counts (percentages) for categorical variables. Continuous variables were compared using the Kruskal–Wallis test; categorical variables were compared using Pearson’s chi-squared test or Fisher’s exact test, as appropriate. * *p* < 0.05 was considered statistically significant.

**Table 2:** Histopathological characteristics of uterine tumors.

Histopathological Diagnosis	Myoma Group (n, %)	Sarcoma/STUMP Group (n, %)
Leiomyoma	80 (88.9%)	-
Leiomyoma + adenomyosis	7 (7.8%)	-
Other benign findings	3 (3.3%)	-
Leiomyosarcoma	-	2 (11.8%)
Carcinosarcoma	-	3 (17.6%)
ESS	-	2 (11.8%)
Adenosarcoma	-	1 (5.9%)
STUMP	-	8 (47.1%)
Other *	-	1 (5.9%)

Data are presented as counts (n) and percentages (%). Categorical variables were compared using Pearson’s chi-squared test or Fisher’s exact test, as appropriate. ESS: endometrial stromal sarcoma; STUMP: smooth muscle tumor of uncertain malignant potential; * Leiomyomas and focal sarcomatous transformation in the background of adenomyoma.

**Table 3:** Surgical management in myoma and sarcoma groups.

Surgical Procedure	Myoma (n, %)	Sarcoma/STUMP (n, %)
Myomectomy	43 (47.7%)	4 (23.5%)
Hysterectomy ± unilateral oophorectomy	3 (3.33%)	4 (23.5%)
Subtotal hysterectomy ± oophorectomy	15 (16.6%)	3 (17.6%)
THBSO	29 (32.2%)	6 (35.3%)

Data are presented as counts (n) and percentages (%). THBSO: total hysterectomy with bilateral salpingo-oophorectomy.

**Table 4:** Serum galectin-1 and MUC1 concentrations according to histological group.

Biomarker	Healthy (n = 49)	Myoma (n = 90)	STUMP (n = 8)	Uterine Sarcoma (n = 9)	Overall *p*-Value
**MUC1 (U/mL)**	14.8 (7.8)	14.0 (9.78)	10.9 (7.85)	24.6 (12.4)	0.031 *
**Galectin-1 (pg/mL)**	11,074 (4874)	12,460 (5835)	13,048 (3344)	20,440 (9665)	<0.001 *

Data are presented as median (interquartile range, IQR). Overall differences were assessed using the Kruskal–Wallis test. * *p* < 0.05 was considered statistically significant.

**Table 5:** Diagnostic performance of galectin-1 and MUC1 across study group comparisons.

Comparison	Biomarker	AUC (95% CI)	Optimal Cutoff	Sensitivity (%)	Specificity (%)
Myoma vs. Sarcoma/STUMP	Galectin-1	0.693 (0.559–0.826)	12,866.4 pg/mL	82.4	53.3
	MUC1	0.618 (0.468–0.768)	9.31 U/mL	94.1	32.2
Healthy vs. Sarcoma/STUMP	Galectin-1	0.785 (0.649–0.920)	17,928 pg/mL	47.1	100.0
	MUC1	0.588 (0.412–0.763)	24.2 U/mL	29.4	98.0
Healthy vs. Myoma	Galectin-1	0.594 (0.496–0.692)	17,509.0 pg/mL	17.8	100.0
	MUC1	0.564 (0.467–0.661)	8.22 U/mL	26.7	91.8
Sarcoma/STUMP vs. Healthy/Myoma	Galectin-1	0.725 (0.597–0.853)	12,866.4 pg/mL	82.4	56.1
	MUC1	0.607 (0.455–0.760)	24.2 U/mL	29.4	93.5

**Table 6:** Exploratory multivariable logistic regression analyses of factors associated with sarcoma/STUMP status.

Variable	OR	95% CI	*p*-Value
Galectin-1 (per 1000 pg/mL)	1.07	1.02–1.13	0.007
Age (years)	0.99	0.92–1.07	0.84
Menopause (yes vs. no)	1.37	0.26–6.98	0.71
MUC1 (per 1 U/mL)	1.05	0.98–1.12	0.145
Age (years)	1.00	0.93–1.07	0.898
Menopause (yes vs. no)	1.26	0.25–5.95	0.773

## Data Availability

The raw data supporting the conclusions of this article will be made available by the authors on request.

## Appendix A

**Table A1:** Internal bootstrap validation of the diagnostic performance of galectin-1 and MUC1 for discriminating leiomyoma from sarcoma/STUMP.

Biomarker	Bootstrap-Validated AUC (95% Interval)	Mean Cutoff (95% Interval)	Sensitivity, % (95% Interval)	Specificity, % (95% Interval)
Galectin-1	0.697 (0.506–0.875)	14,430 (11,431–17,994) pg/mL	60.7 (14.3–100.0)	61.0 (30.0–90.3)
MUC1	0.620 (0.421–0.826)	14.77 (9.10–23.80) U/mL	57.3 (0–100.0)	56.2 (20.7–94.1)
